# Is an Impacted Tooth Per Se an Indication for Operation?

**Published:** 1920-04

**Authors:** Leon Harris


### IS AN IMPACTED TOOTH PER SE AN INDICATION FOR OPERATION?

BY LEON HARRIS, M.D., D.D.S.

In the July issue of *Dental Items of Interest* appears an article from the pen of Dr. A. Berger, which should be of grave concern to the dental and medical professions and should stimulate a discussion that ought once and for all to settle the indications for removal of impacted teeth.

Dr. Berger's article in itself is a reply to that of Dr. McCoy, who, speaking on the subject, counsels that— "if such a condition is unaccompanied by any pathological symptoms and the patient was not anxious to have the work done, I would let sleeping dogs lie." Taking up the pen against the above advice of Dr. McCoy, one would naturally look for more valid arguments on the part of Dr. Berger for his more aggressive surgical procedure than are presented in his article.

Dr. Berger takes issue with a great number of practitioners who "are laboring under the erroneous impression that the impaction or malposition of teeth is dependent solely upon some incidental environmental condition" and in turn tells us that "these conditions may be more justly regarded as forms of abnormal dentition which most likely have some ontogenetic significance, or are caused by some obscure developmental abnormality," which adds about as much information to our conception of the subject as the statement which he criticises. The point is that very little is known about the etiology of impacted teeth, nor does the etiology in this case help us much in our treatment, for it can not be corrected. To theorize on a proposition makes one guess as valuable as another, for the one who thinks that it is due to an incidental environmental condition adds as much to the general store of knowledge as the other that is due to an obscure developmental abnormality.

What is important for us to consider, and what I had hoped to see Dr. Berger bring out with clearness and conviction, is that impacted teeth should be removed without any provocation. When he says that they should be removed upon reasonable indication he can not take issue with Dr. McCoy, for "a sleeping dog" offers no indication for attack, but the very fact that he takes issue with Dr. McCoy leads us to believe that Dr. Berger would remove any impacted tooth from the mere fact of its being impacted, although he is very wary in his statements and does not say so directly; but at least that is the implication.

### DR. HARRIS'S VIEWS

Now, I do not wish to go on record as begging the question. I will not make the statement that impacted teeth should often be removed without any provocation. If often, then why not always? I will state most emphatically that there must be a clear-cut indication for the removal of an impacted tooth, such as reflex neuralgic pain, suppuration and absorption. If we go on the assumption that an impacted tooth may at some future time be the seat of pathologic changes, than a normally erupting third molar is subject to the same conditions, and should be removed. Dr. Berger evidently argues his case from the point of view of prevention. The failure to remove such teeth has often led to suppuration, cyst formation, and reflex disturbances. But how about the thousands of persons who have impacted teeth and find themselves in perfect health? Are we to subject all such patients to the mutilation of a serious operation, with the tremendous after effects and suffering, purely in the interests of prophylaxis? If so, then why should not we all have our appendices removed? Dr. Berger will readily grant that the appendix is both a superfluous and an insidious organ. More people who are in perfect health today will find themselves suffering from an attack of appendicitis that will necessitate the hurried removal to a hospital tomorrow, and by the time they will be put upon the operating table, a good many appendices will be found to be gangrenous, a good many cases will have developed peritonitis and a good many others will never leave the operating table alive. Surely, this offers a much more gloomy picture of morbidity and mortality than can possibly follow the neglect to remove an impacted tooth; and to think that all this might have been prevented by the removal of the appendix before any sign of symptoms! And yet people do not rush to surgeons to have their appendices removed.

How about the tonsils? Tonsilitis, scarlet fever, diphtheria, quinsy, rheumatism and endocarditis are conceded by the best authorities to follow diseases of the tonsils, and yet the indiscriminate removal of the tonsils, without a decided indication for it, but merely for the sake of prevention, is not countenanced by the medical profession. "*Nolle me nocere*" is a dictum that has survived the devastating hand of time and surgical aggressiveness, and sways the hand of the conscientious surgeon today as much as it ever did.

Let me take the case of a young woman who has a surviving temporary cuspid with normal lateral and bicuspids and the permanent cuspid transversely placed above these teeth without any disturbing symptoms. Would Dr. Berger place this case among those that should be "often removed without provocation," or would that case be outside of this pale? A cuspid thus placed can hardly be removed without compromising some of the permanent teeth and without the sacrifice of the temporary cuspid. Would all that destruction of tissue be indicated in a symptomless case, and merely from a prophylactic point of view? No; and decidedly, no! Where such a case has any symptomatology it assumes quite a different aspect. For the relief of symptoms no sacrifice is too great. But to argue simply upon a surgical premise that anything abnormal and potential of ill results must yield to the surgeon's knife, even though such potentialities are not definite and constant, will bring upon the oral surgeon the very stigma that he is most anxious to avoid.

The case of impacted teeth is far different from those of incipient, malignant diseases where experience has taught us that a bad prognosis is inevitable unless there is a complete eradication before it has become firmly rooted. The case of Mrs. A. K., which Dr. Berger quotes in support of his argument for the indiscriminate removal of impacted third molars, carries no conviction, for the following reasons: first, because one swallow does not make a summer: secondly, because Dr. Berger himself says that following the removal of the second molar the patient had a remission of her symptoms until about "two months ago." It will be a considerable time following the removal of her third molars before there can be any definite relation between cause and effect.

In this connection, I quote a very similar case of a woman who came to this city from Boston, complaining of a similar lumbar pain that had lasted over a year and for which eminent gynecologists, both here and in Boston, had failed to provide any relief. Finding a few infected teeth in her mouth I urged their removal, and this was followed by a complete remission of all symptoms. Evidently her trouble was not in the teeth *per se*, but in the absorption of toxins. Similarly, impacted teeth *per se* are not in themselves productive of disturbances, but the fact is that they are often the seat of infection or give rise to pressure symptoms. Unless, therefore, there are unmistakable symptoms of pathologic changes, I would, like Mr. McCoy, let "sleeping dogs lie."—*Dental Items of Interest*, November, 1919.

---

### OUR DUTY TO THE PUBLIC

Dr. E. Kells, in the National Dental Meeting last summer, made an urgent appeal to have every person who was threatened with toothache to take care of it without delay. The dental profession is to blame because the people don't realize their dangers. If we want to do something worth while we should make up a good big sign that everyone will see saying something like this: *You must not let your teeth ache and you should learn why!*

